# Outcomes of Physician-Modified Endografts for Juxtarenal and Pararenal Abdominal Aortic Aneurysms: A Systematic Review and Single-Arm Meta-Analysis

**DOI:** 10.3390/jcm15176578

**Published:** 2026-08-26

**Authors:** Haoran Wang, Mohammed Al-Falahi, Bernhard Hruschka, Panagiotis Doukas, Christian Uhl, Jelle Frankort, Alexander Gombert

**Affiliations:** Department of Vascular Surgery, Medical Faculty, RWTH Aachen University, 52074 Aachen, Germany; hawang@ukaachen.de (H.W.);

**Keywords:** physician-modified endograft, PMEG, juxtarenal abdominal aortic aneurysm, pararenal abdominal aortic aneurysm, fenestrated EVAR, meta-analysis

## Abstract

**Background:** To present perioperative and follow-up outcomes of physician-modified endograft (PMEG) repair for juxtarenal and pararenal abdominal aortic aneurysms. **Methods:** This PRISMA 2020 systematic review and single-arm meta-analysis was registered with PROSPERO (CRD420261340339). PubMed, Embase, Web of Science, the Cochrane Library, and ClinicalTrials.gov were searched until December 2025. Eligible reports described full-text-confirmed cohorts of at least five patients treated with PMEG. The strict primary analysis required endpoint-separable juxtarenal or pararenal populations and used random-effects generalized linear mixed models for pooled proportions. Methodological quality and evidence certainty were assessed using the Joanna Briggs Institute checklist and GRADE framework. **Results:** Fifteen eligible reports described 981 patients before overlap adjudication; 10 reports originated from one institution and contained overlapping populations. After pathology and endpoint-level overlap adjudication, one to four independent populations informed each endpoint estimate, and single-source outcomes were not pooled. Technical success was 97.8% (95% CI, 94.8–99.1%; three populations, 228 patients), and 30-day mortality was 2.3% (95% CI, 1.1–4.8%; four populations, 299 patients). Acute kidney injury, stroke, and spinal cord ischemia occurred in 4.5%, 1.6%, and 1.7% of patients, respectively. During available follow-up, the single-source Type III endoleak estimate was 3.4% (7/203), any reintervention was 13.2% (95% CI, 2.9–43.7%; two populations, 217 patients; I^2^ = 89.0%), and patient-level target-vessel occlusion was 2.4% (95% CI, 0.6–9.1%; 2 populations, 83 patients). Follow-up estimates were exploratory report-period proportions. GRADE certainty was very low for all key outcomes. **Conclusions:** PMEG repair demonstrated a high technical-feasibility signal and low observed early event rates in experienced centers, but clinical effectiveness and durability remain uncertain. The evidence base is dominated by overlapping expert-center reports and should not be interpreted as establishing equivalence or superiority to custom-manufactured FEVAR.

## 1. Introduction

Complex abdominal aortic aneurysms that extend to, or closely approach, the renal and visceral aortic segments remain difficult to repair endovascularly. Standard infrarenal endografts require an adequate proximal neck and cannot reliably seal in this anatomy. Juxtarenal and pararenal abdominal aortic aneurysms are defined by an inadequate infrarenal neck, close proximity to the renal arteries, or involvement of the renal artery origins, and together account for an estimated 15% to 20% of abdominal aortic aneurysms requiring repair [1,2]. Open surgical repair remains an established option, but many affected patients are elderly, medically frail, or at increased operative risk. Conventional infrarenal endovascular aneurysm repair is often anatomically unsuitable [2,3]. Custom-manufactured fenestrated and branched endovascular devices can extend the proximal sealing zone while preserving renal and visceral perfusion. Their use, however, may be constrained by manufacturing lead time, restricted availability, reimbursement or regulatory barriers, and patient-specific anatomy, especially in urgent or non-deferrable settings.

Physician-modified endografts (PMEGs) offer an off-label, anatomy-tailored strategy for complex endovascular aortic repair in centers with established expertise. In this approach, commercially available endografts are modified on the back table to create patient-specific fenestrations or scallops, avoiding the manufacturing delay required for custom devices [4]. This flexibility may be clinically important for symptomatic aneurysms, contained or impending rupture, rapid aneurysm enlargement, proximal seal failure after previous endovascular repair, or patients who are poor candidates for open repair and lack a standard endovascular option. PMEG repair nevertheless remains technically demanding and operator-dependent. Outcomes may vary with case selection, preoperative planning, modification technique, intraoperative imaging, target-vessel incorporation, and postprocedural surveillance.

The evidence base for PMEG has grown, but its interpretation remains difficult. Many reports combine juxtarenal, pararenal, paravisceral, suprarenal, and thoracoabdominal pathologies, despite differences in anatomic complexity, target-vessel burden, aortic coverage, spinal cord ischemia risk, and durability expectations. Outcome definitions and denominators are also inconsistent, particularly for technical success, renal outcomes, endoleak subtypes, target-vessel patency, patient-level versus vessel-level reporting, and reintervention. Several publications also derive from overlapping institutional experiences, creating a risk of duplicate patient counting when reports are pooled at the publication level without full-text adjudication. Previous reviews have therefore provided useful broad summaries of PMEG practice, but their findings may be less directly applicable to juxtarenal and pararenal abdominal aortic aneurysm repair specifically [5].

This systematic review and single-arm meta-analysis aims to present perioperative and follow-up outcomes of physician-modified endograft repair specifically for patients with juxtarenal and pararenal abdominal aortic aneurysms.

## 2. Materials and Methods

### 2.1. Protocol and Registration

This systematic review was conducted in accordance with the Preferred Reporting Items for Systematic Reviews and Meta-Analyses (PRISMA) 2020 guidelines [6]. The protocol was registered with PROSPERO (CRD420261340339). The completed PRISMA 2020 checklist is provided in Supplementary File S1.

### 2.2. Information Sources

Searches were last updated on 26 December 2025, in five electronic sources: PubMed (*n* = 770), Embase via Ovid (*n* = 959), Web of Science Core Collection (*n* = 573), Cochrane Library (*n* = 1), and ClinicalTrials.gov (*n* = 25). No language or date restrictions were applied. Reference lists of included reports and relevant review articles were hand-searched for additional eligible records.

### 2.3. Search Strategy

The search strategy combined population terms (abdominal aortic aneurysm, juxtarenal, pararenal, suprarenal) with intervention terms (physician-modified endograft, PMEG, fenestrated endovascular repair, homemade, physician-modified). Boolean operators and Medical Subject Headings (MeSHs) or Emtree terms were used where applicable. The full electronic search strategy for each database is provided in Supplementary File S2.

### 2.4. Study Selection Process

Records were imported into Zotero (version 8; Corporation for Digital Scholarship, Vienna, VA, USA) and deduplicated. From the 2303 database citations, 1033 duplicates were removed, yielding 1270 unique records. ClinicalTrials.gov records were screened separately and did not identify additional eligible clinical reports beyond the published reports. Title and abstract screening was performed independently by H.W. and H.Z., and 61 records underwent full-text assessment. Disagreements were resolved by consensus.

### 2.5. Eligibility Criteria

Reports were eligible if they described patients with juxtarenal or pararenal abdominal aortic aneurysm pathology confirmed at full-text review, used back-table physician-modified fenestrated or branched endografts, and reported at least one extractable clinical outcome. For this review, juxtarenal aneurysms were those extending to or closely approaching the renal arteries without an adequate infrarenal sealing neck, whereas pararenal aneurysms involved at least one renal artery but did not extend into the superior mesenteric or celiac segment. Paravisceral, suprarenal, and thoracoabdominal aneurysms were outside the target population unless a juxtarenal or pararenal subgroup could be separated. Original clinical reports with at least five patients were included to avoid reliance on isolated case reports while retaining the limited non-University of Washington evidence base. Bench experiments, feasibility-only reports, cost analyses, reports describing in situ laser fenestration only, and case reports were excluded. Comparator reports were not excluded a priori. When a comparator report described a distinct PMEG arm, only the PMEG arm was extracted, provided it could be isolated without mixing a non-target population.

### 2.6. Data Extraction

H.W. and H.Z. independently extracted data using a standardized form. Discrepancies were resolved by discussion and consensus. Full texts were reassessed record by record, with attention to population definition, intervention type, outcome availability, sample size, and table-level evidence. Reports containing at least some relevant juxtarenal or pararenal cases were retained for report-level characterization. For the strict primary quantitative synthesis, however, an endpoint was used only when its denominator represented an entirely target-segment cohort or when the target subgroup could be separated. Mixed cohorts with inseparable infrarenal, paravisceral, thoracoabdominal, penetrating aortic ulcer, or post-endovascular seal-failure pathology were retained only in a broader mixed-pathology sensitivity analysis.

Report-level variables included publication year, design, investigational device exemption (IDE) status, center type, sample size, age, sex distribution, aneurysm type, device platform, fabric-opening method, fenestration reinforcement, target-vessel count, urgent-case eligibility, postoperative endograft infection, and follow-up duration. Outcome extraction focused on binary event counts and denominators attributable to prespecified perioperative or follow-up endpoints. The complete extracted dataset is provided in Supplementary Data File S1, and pathology and modification-method audits are provided in Supplementary Tables S6 and S7.

### 2.7. Handling of Overlapping Cohorts

Because several eligible reports originated from the same institutional PMEG program, the unit of analysis was the independent patient population rather than the individual publication. Potential overlap was adjudicated using institution, author group, trial registration or IDE status, enrollment period, sample size, baseline characteristics, device platform, and report objective. Reports judged to represent serial updates, subset analyses, or endpoint-specific analyses of a common underlying cohort were grouped into a single overlap cluster.

Reports from the University of Washington PMEG program were assessed separately because they accounted for a substantial proportion of the available evidence [7,8,9,10,11,12,13,14,15,16]. Starnes 2012 was treated as an independent pre-IDE retrospective cohort because it included patients treated before initiation of the subsequent physician-sponsored IDE trial [7]. Reports published from 2013 onward were classified as serial, subset, or endpoint-specific analyses of the UW IDE/NCT01538056 cohort. The full adjudication process and endpoint-level data use are detailed in Supplementary Table S2.

For the primary meta-analysis, no more than one report from the UW IDE overlap cluster was allowed to contribute to any pooled endpoint. Report selection was performed at the endpoint level. For perioperative outcomes, priority was given to the report with the largest extractable denominator, a compatible endpoint definition, and a clearly specified time point. For outcomes reported during available follow-up, follow-up duration, denominator clarity, and outcome specificity were also considered. Overlapping reports not used for a pooled endpoint were retained for report characterization, overlap assessment, endpoint mapping, and narrative synthesis.

This overlap-management approach was developed after full-text review revealed substantial serial and subset reporting from a single institutional program. It is therefore reported as a deviation from the original PROSPERO protocol. The rationale for each selected UW endpoint source and the feasibility of replacing it with the latest comprehensive UW report are provided in Supplementary Tables S2 and S4.

### 2.8. Outcomes

Prespecified perioperative outcomes included technical success, 30-day mortality, acute kidney injury, stroke, spinal cord ischemia, Type Ia endoleak, Type III endoleak, any endoleak, and target-vessel occlusion at 30 days. Follow-up outcomes included Type Ia endoleak, Type III endoleak, any endoleak, target-vessel occlusion, and any reintervention during available follow-up. Technical-success definitions were extracted at the component and reporting-level scale and were not identical across reports. Most shared core elements of successful device delivery or deployment, preservation of intended target vessels or components, and absence of major Type I or III endoleak, but reporting windows ranged from procedural completion to 30 days. Patient-level estimates with clinically compatible core components were used in the primary analysis; a sensitivity analysis was restricted to reports with explicit composite definitions. The Tachida 2023 [13] definition most closely reflected Society for Vascular Surgery reporting standards but was not treated as a universal definition for all reports. Definitions of other outcomes, including acute kidney injury, stroke, and spinal cord ischemia, were accepted as reported by the original investigators because no uniform criterion was applied across all source reports.

Patient-level denominators were used for most outcomes. Thirty-day target-vessel occlusion was analyzed at the vessel level because only the number of initially preserved branch vessels was available. During available follow-up, patient-level target-vessel occlusion was reconstructed from reports that stated the number of patients with at least one occluded target vessel. Index failure to cannulate or preserve a target vessel was classified as technical or incorporation failure rather than as postprocedural occlusion. A broader sensitivity analysis combined index target-vessel loss with postprocedural loss of patency. Outcomes were pooled when at least two independent populations contributed clinically comparable data. Single-source outcomes were reported with exact confidence intervals and were not presented as pooled estimates.

### 2.9. Methodological Quality and Certainty and Evidence Certainty Assessment

Methodological quality was assessed at the report level using the Joanna Briggs Institute Critical Appraisal Checklist for Case Series [17]. Because several reports originated from overlapping University of Washington/Harborview IDE/NCT01538056 populations, these assessments were interpreted as report-level appraisals rather than as assessments of independent patient cohorts (Supplementary Table S5). Certainty of evidence for prespecified key outcomes was assessed using the Grading of Recommendations Assessment, Development and Evaluation (GRADE) framework [18]. The initial certainty rating for all outcomes was low because the included reports were observational and noncomparative. Certainty was further adjusted for methodological limitations, inconsistency, indirectness, imprecision, and publication bias, with potential upgrading for large effect size.

### 2.10. Statistical Analysis

All analyses were performed in R (version 4.5.2; R Foundation for Statistical Computing, Vienna, Austria) using the meta package (version 8.2.1). Pooled proportions were estimated in a single-arm meta-analysis using random-effects generalized linear mixed models (GLMMs) with a logit link and maximum-likelihood estimation of between-population variance. For endpoints with zero events in all contributing populations, an exact binomial confidence interval was calculated over the aggregated all-zero denominator and heterogeneity was reported as not estimable. A single contributing population was summarized using an exact Clopper–Pearson estimate and was not pooled. Heterogeneity was quantified using I^2^, but an observed I^2^ of 0% was not interpreted as evidence of homogeneity when only two to four independent populations contributed [19].

Timepoints were standardized to 30 days and during available follow-up. Because follow-up durations, Kaplan–Meier estimands, and available risk sets were incompatible, a valid pooled 1-year analysis could not be constructed. During-follow-up, proportions were therefore treated as exploratory report-period estimates rather than as estimates of a common fixed-time cumulative incidence. Sensitivity analyses examined the submitted mixed-pathology dataset, all eligible reports without overlap adjustment, explicit technical-success definitions, replacement of Tachida 2023 [13] with the percentage-derived Starnes 2024 [14] technical-success estimate, exclusion of the inferred-zero acute kidney injury row, broader target-vessel loss classification, and leave-one-out source influence. Subgroup analyses by IDE status, device platform, modification type, and number of target vessels were not retained because too few independent populations were available. Formal small-study effect testing was planned only for outcomes with at least 10 independent populations; no primary endpoint met this threshold. No original report authors were contacted for missing data.

Pooled results are reported as summary proportions with 95% confidence intervals, together with the number of contributing independent populations and the pooled denominator. Because many endpoints were reported by only a small number of independent populations, interpretation emphasized effect size, precision, and clinical coherence rather than statistical significance alone. Estimates based on only two independent populations, all during available-follow-up analyses, and all single-source estimates were considered exploratory.

## 3. Results

### 3.1. Study Selection Results

**Figure 1:**
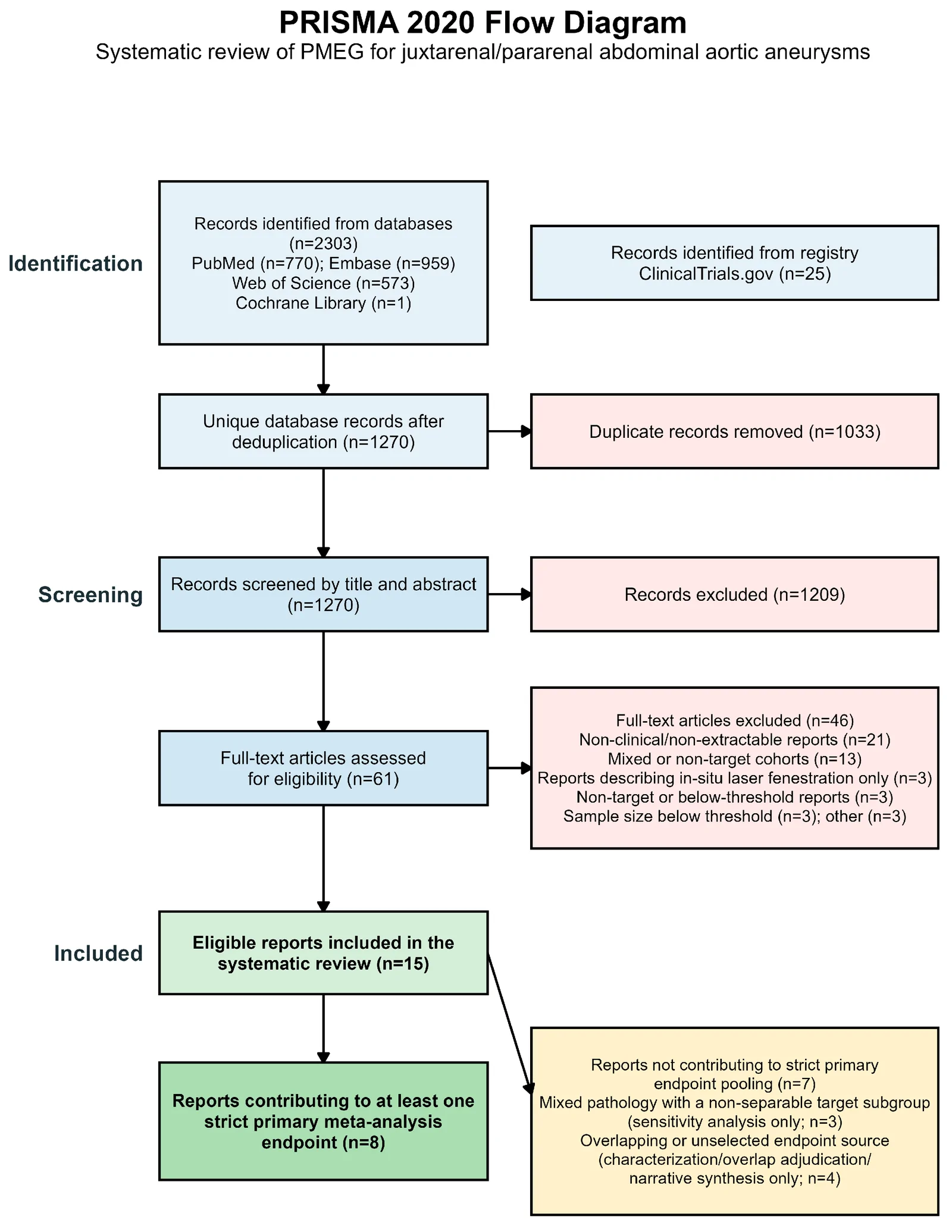
PRISMA flow diagram. A total of 2303 records were identified from four electronic databases, and 25 ClinicalTrials.gov records were screened separately. After removal of 1033 duplicates, 1270 database records were screened and 61 underwent full-text assessment. Fifteen reports were included in the systematic review; eight contributed to at least one strict primary endpoint.

A total of 2303 records were identified from database searches (PubMed 770, Embase 959, Web of Science 573, Cochrane Library 1), with 25 additional records from ClinicalTrials.gov. After removal of 1033 duplicate database records, 1270 unique records were screened by title and abstract. ClinicalTrials.gov records were screened separately and did not yield additional eligible reports. Sixty-one records proceeded to full-text assessment. Forty-six full-text articles were excluded; detailed reasons are provided in Figure 1 and Supplementary Table S1. Fifteen reports were included in the systematic review. Eight reports contributed to at least one strict primary endpoint. Three mixed-pathology reports contributed only to sensitivity analyses, and four overlapping or unselected endpoint reports were retained for characterization, overlap adjudication, or narrative synthesis.

### 3.2. Report Characteristics

The 15 included reports were published between 2012 and 2025 and described 981 reported patients before overlap adjudication (Table 1) [7,8,9,10,11,12,13,14,15,16,20,21,22,23,24]. Five reports collected data prospectively under an investigational device exemption (IDE) protocol; 10 were retrospective analyses. Nine reports were conducted under an IDE and six were non-IDE reports. Fourteen reports were single-center and one was multicenter. Back-table modification was explicitly described in 12 reports and was not specified in three.

Ten of 15 reports originated from the University of Washington [7,8,9,10,11,12,13,14,15,16]. Starnes 2012 [7] was treated as an independent pre-IDE University of Washington cohort. Subsequent University of Washington reports published from 2013 onward were treated as serial, subset, or endpoint-specific analyses of the IDE/NCT01538056 cohort. Sample sizes within the University of Washington IDE/NCT01538056 reports ranged from 26 to 203 PMEG-treated patients, with some outcome-specific denominators differing from the treated cohort denominator. The remaining five reports came from independent international centers in Switzerland, Korea, Hungary, Germany, and Spain, with sample sizes ranging from five to 37 patients [20,21,22,23,24]. Lescan 2025 was the only five-patient report; the smallest independent non-UW report contributing to the strict primary analysis was Csobay-Novák 2025, with 11 patients [22,23].

Reported mean or median age ranged from 74 to 82 years, and cohorts were predominantly male. Follow-up duration ranged from approximately 10 to 59 months among reports that provided this information. Follow-up was not reported in five reports, and loss to follow-up was reported in only two of 15 reports. Device platforms included Cook, Terumo, and Medtronic, with several larger series using multiple platforms. Full-text pathology adjudication identified three reports with inseparable mixed denominators: Kim 2024 included 10 juxtarenal, one infrarenal, and one thoracoabdominal aneurysm; Lescan 2025 included one pararenal aneurysm, two post-EVAR Type Ia seal failures, and two penetrating aortic ulcers; and Álvarez Marcos 2025 included six explicitly juxtarenal or pararenal aneurysms among 18 complex abdominal cases [21,23,24]. These reports remained eligible for qualitative characterization but were excluded from strict primary pooling and retained in a mixed-pathology sensitivity analysis (Supplementary Table S7).

### 3.3. Modification Techniques, Infection, and Urgent-Case Selection

The fabric-opening method could be identified in 12 reports: nine described the method directly and three referred to an earlier published PMEG technique. Most used ophthalmic, electrosurgical, or low-temperature cautery, whereas Chan 2023 used a scalpel [7,8,9,10,12,13,14,20,21,22,23,24]. No included back-table PMEG report used laser fenestration. In Csobay-Novák 2025, the punch card served as a positioning template rather than the cutting instrument, and the graft fabric was opened using sterile cautery [22]. Fenestrations were generally reinforced with a radiopaque snare or wire secured by suture; Csobay-Novák 2025 [22] used a closed-ring reinforcement. In Starnes 2024, the seven reported Type III endoleaks comprised six Type IIIc renal stent separations and one Type IIIa limb-component separation; none was attributed by the investigators to the fabric-opening instrument [14]. Because method-stratified Type III endoleak denominators were unavailable, a causal or comparative analysis of modification methods was not possible (Supplementary Table S6).

Open back-table exposure and manipulation of the graft fabric create a theoretical concern regarding contamination and delayed graft infection. One presumed endograft infection was reported at 750 days in the Starnes 2024 UW IDE cohort; the unconfirmed infection described in the overlapping Murphy 2025 subset likely represented the same patient and was not counted as a second independent event [14,15]. Most reports did not define systematic postoperative infection surveillance, and no additional graft infection was identified in reports that explicitly addressed this outcome, precluding quantitative synthesis. Infection status at treatment, such as mycotic aneurysm or infectious penetrating aortic ulcer, was recorded separately from postoperative graft infection.

No report provided a complete denominator and outcomes for all urgent patients assessed but found ineligible for PMEG. Csobay-Novák 2025 excluded hemodynamically unstable patients requiring immediate repair, and Kim 2024 treated only hemodynamically stable patients, but neither reported the number excluded [21,22]. Starnes 2024 described 228 enrolled patients, 217 who completed screening, 205 in whom implantation was initiated, and 203 who received PMEG, but screen failure and withdrawal were not stratified by urgent presentation [14]. These missing screened-but-untreated denominators limited assessment of selection bias in urgent practice.

### 3.4. Thirty-Day Outcomes

**Figure 2:**
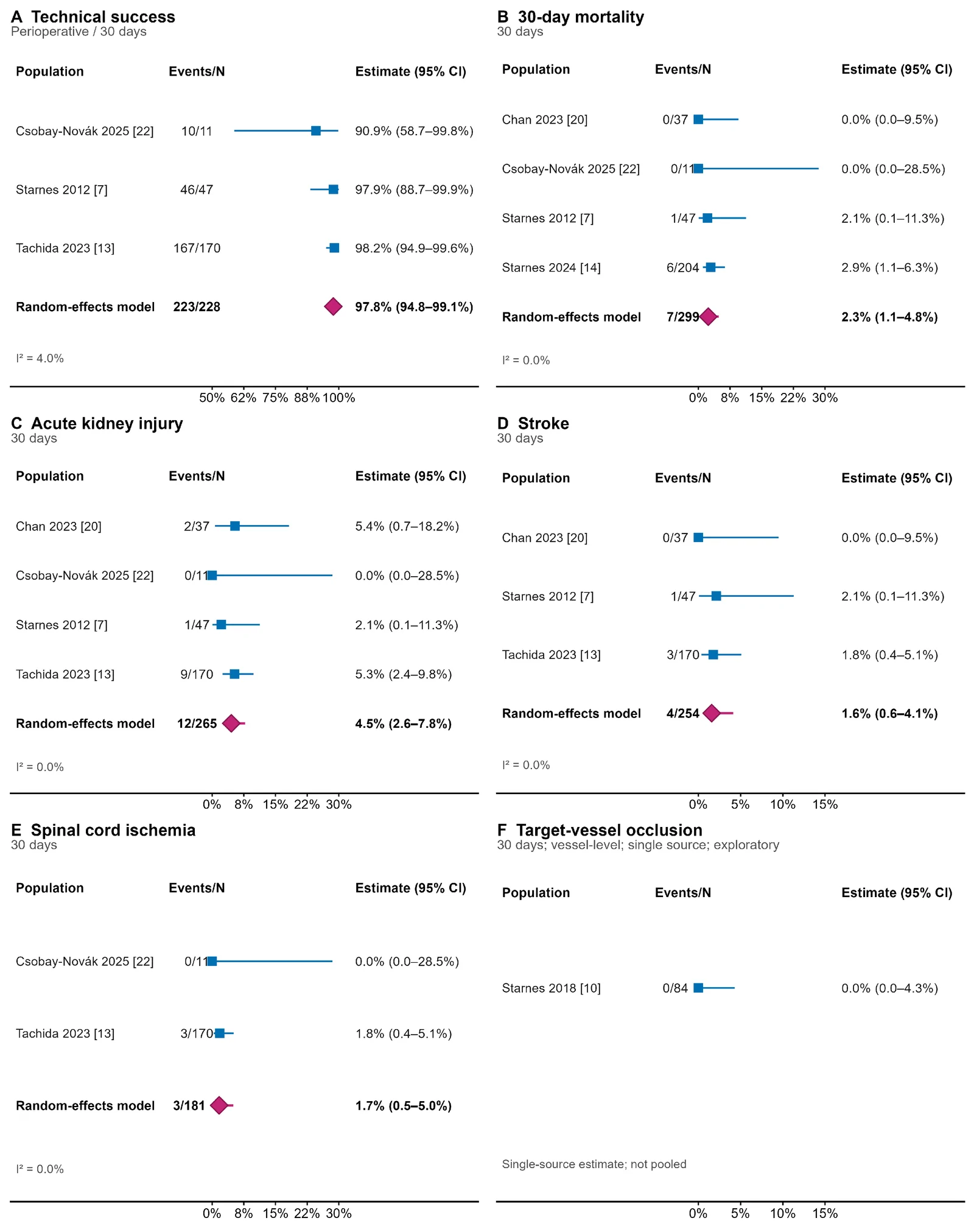
Perioperative forest plots from the strict overlap- and pathology-adjusted analysis. (**A**) Technical success (three independent populations, 228 patients). (**B**) 30-day mortality (4 independent populations, 299 patients). (**C**) Acute kidney injury (4 independent populations, 265 patients). (**D**) Stroke (three independent populations, 254 patients). (**E**) Spinal cord ischemia (2 independent populations, 181 patients). (**F**) Target-vessel occlusion, vessel-level denominator (single-source estimate, 0/84 vessels; not pooled; exploratory). Squares represent study estimates, lines represent 95% confidence intervals, and diamonds represent pooled estimates [7,10,13,14,20,22].

In the strict overlap- and pathology-adjusted analysis, technical success was 97.8% (95% CI 94.8–99.1%; three independent populations, 228 patients; I^2^ = 4.0%) (Figure 2A). Thirty-day mortality was 2.3% (95% CI 1.1–4.8%; four independent populations, 299 patients; I^2^ = 0.0%) (Figure 2B). The mortality denominator included Starnes 2024 as 6/204, as reported for that endpoint, although 203 patients received PMEG [14].

Acute kidney injury was 4.5% (95% CI 2.6–7.8%; four independent populations, 265 patients; I^2^ = 0.0%) (Figure 2C). Stroke was 1.6% (95% CI 0.6–4.1%; three independent populations, 254 patients; I^2^ = 0.0%) (Figure 2D), and spinal cord ischemia was 1.7% (95% CI 0.5–5.0%; two independent populations, 181 patients; I^2^ = 0.0%) (Figure 2E). These low observed I^2^ values should not be interpreted as evidence of homogeneity because only two to four independent populations informed each estimate.

No Type Ia or Type III endoleak was observed among the 41 patients contributing to either 30-day endpoint; the exact estimates were 0.0% (95% CI 0.0–8.6%; two independent populations), and heterogeneity was not estimable. Any endoleak at 30 days was 17.1% (95% CI 10.0–27.8%; two independent populations, 70 patients; exploratory). Thirty-day target-vessel occlusion was available from one strict source and was not pooled. Starnes 2018 reported no loss of patency among 84 initially preserved branch vessels, corresponding to 0.0% (95% CI 0.0–4.3%; vessel-level; exploratory) (Figure 2F) [10]. The 1/43 vessel event in Csobay-Novák 2025 was an index accessory renal incorporation failure rather than a documented 30-day occlusion; including it in a broader target-vessel loss sensitivity analysis yielded 0.8% (95% CI 0.1–5.4%; two populations, 127 vessels) [10,22].

Denominators differed because endpoint reporting was not uniform. The 30-day any-endoleak denominator comprised Starnes 2017 (10/59) and Csobay-Novák 2025 (2/11) [9]. Acute kidney injury included four sources; Csobay-Novák 2025 [22] was coded as 0/11 based on no major adverse event and no renal-function decline rather than a separately tabulated acute kidney injury endpoint (Supplementary Table S2).

Mortality events came from the independent pre-IDE Starnes 2012 cohort (1/47) and the retained UW IDE source, Starnes 2024 (6/204), whereas Chan 2023 and Csobay-Novák 2025 reported no 30-day deaths [7,14,20,22]. Accordingly, the mortality estimate was based on only seven events despite its apparently narrow interval and low observed heterogeneity.

### 3.5. Outcomes During Available Follow-Up

**Figure 3:**
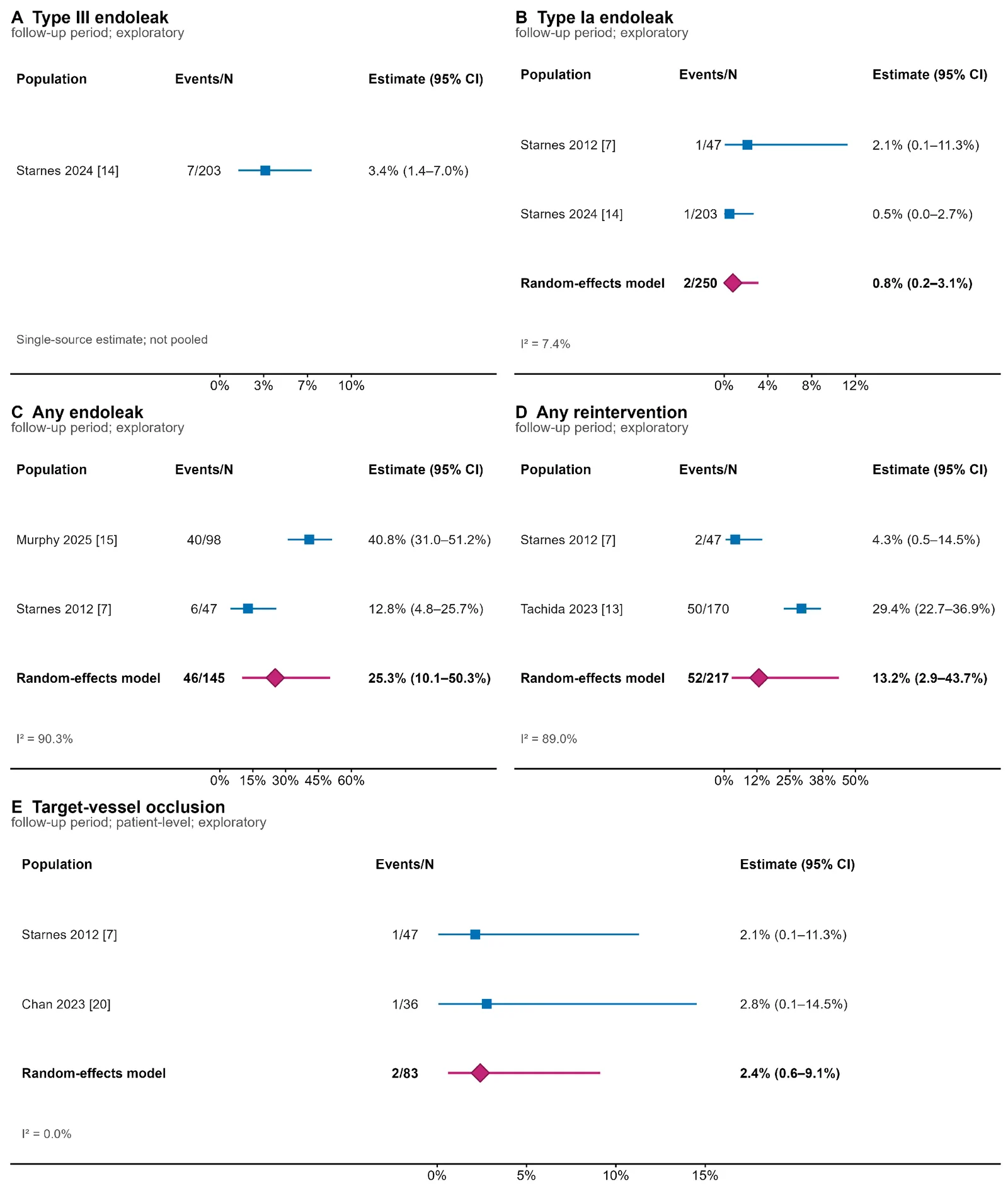
Exploratory forest plots during available follow-up. (**A**) Type III endoleak (single-source estimate, 7/203; not pooled). (**B**) Type Ia endoleak (2 independent populations, 250 patients). (**C**) Any endoleak (2 independent populations, 145 patients). (**D**) Any reintervention (2 independent populations, 217 patients). (**E**) Target-vessel occlusion, patient-level denominator (2 independent populations, 83 patients). These report-period estimates reflect source-specific follow-up and should not be interpreted as a common fixed-time cumulative incidence. Squares represent study estimates, lines represent 95% confidence intervals, and diamonds rep-resent pooled estimates [7,13,14,15,20].

All during-follow-up estimates were exploratory report-period proportions rather than estimates of a common fixed-time cumulative incidence. Type III endoleak was available from one strict target-population source and was therefore not pooled; Starnes 2024 [14] reported 7/203 events, corresponding to 3.4% (95% CI 1.4–7.0%) (Figure 3A). Type Ia endoleak was 0.8% (95% CI 0.2–3.1%; two independent populations, 250 patients; I^2^ = 7.4%) (Figure 3B). Any endoleak was 25.3% (95% CI 10.1–50.3%; two independent populations, 145 patients; I^2^ = 90.3%) (Figure 3C), and any reintervention was 13.2% (95% CI 2.9–43.7%; two independent populations, 217 patients; I^2^ = 89.0%) (Figure 3D). Patient-level target-vessel occlusion was 2.4% (95% CI 0.6–9.1%; two independent populations, 83 patients; I^2^ = 0.0%) based on Starnes 2012 (1/47) and Chan 2023 (1/36) (Figure 3E) [7,20].

The strict any-reintervention analysis comprised Starnes 2012 (2/47) and Tachida 2023 (50/170), for a crude aggregate proportion of 52/217 (24.0%) [7,13]. The GLMM estimate of 13.2% was lower than the crude proportion because the model combined study-specific logits with random effects rather than weighting events and patients as a single cohort; the marked difference between the two source proportions (4.3% and 29.4%) also produced substantial heterogeneity. The wide model-based interval and leave-one-out range indicated that this estimate was source-sensitive and should not be interpreted as a simple cumulative event risk.

A standardized 1-year meta-analysis was not feasible. Tachida 2023 [13] and Starnes 2024 [14] each reported 84% Kaplan–Meier freedom from reintervention at 1 year from the same UW IDE population, without compatible event counts and risk sets, while Chan 2023 reported 91% freedom from secondary reintervention and 96% target-vessel patency using different estimands [13,14,20]. These values were retained for narrative context only. The strict primary results are summarized in Table 2.

### 3.6. Methodological Quality and Certainty of Evidence

Methodological quality was assessed for all 15 eligible reports using the JBI checklist for case series. Thirteen reports were rated as having high methodological quality and two as having moderate quality. The most frequent limitations were incomplete reporting of consecutive and complete inclusion of eligible participants, particularly in retrospective subset analyses and late follow-up reports from the overlapping UW IDE/NCT01538056 cohort (Supplementary Table S5).

GRADE certainty was very low for all prespecified key outcomes, including technical success, 30-day mortality, Type III endoleak, target-vessel occlusion, and reintervention (Supplementary Table S3). Only one to four independent populations informed each strict estimate. Downgrading was driven by noncomparative design, expert-center indirectness, imprecision, and possible publication bias; inconsistency provided an additional basis for downgrading any reintervention. Low observed I^2^ values for sparse early outcomes did not materially increase certainty.

### 3.7. Sensitivity Analyses

The broader overlap-adjusted mixed-pathology sensitivity reproduced the submitted estimates: technical success 97.3% (95% CI 94.5–98.7%; six populations, 263 patients), 30-day mortality 2.1% (95% CI 1.0–4.4%; six populations, 329 patients), acute kidney injury 5.1%, stroke 1.4%, and spinal cord ischemia 2.0%. These results were directionally similar to the strict target-population estimates, although any reintervention during available follow-up changed from 13.2% in the strict analysis to 8.7% (95% CI 2.0–31.3%; four populations, 240 patients; I^2^ = 67.5%) after inclusion of the inseparable mixed-pathology reports.

Within the strict target population, Tachida 2023 was the only report with an explicit composite technical-success definition; its single-source estimate was 98.2% (167/170; exact 95% CI 94.9–99.6%) and was not pooled [13]. The sensitivity restricted to four reports with explicit composite definitions yielded 97.6% (95% CI 94.3–99.0%; four populations, 205 patients), although three of these reports contained inseparable mixed pathology and this analysis addressed definition rather than population specificity. Replacing Tachida 2023 with the larger but percentage-derived Starnes 2024 estimate as the UW IDE technical-success source changed the result from 97.8% to 94.3% (95% CI 90.7–96.5%; three populations, 261 patients) [13,14]. Starnes 2024 could not replace the selected UW source for every endpoint because compatible numerator–denominator pairs were unavailable, particularly for acute kidney injury, stroke, spinal cord ischemia, any reintervention, and target-vessel occlusion [14].

Target-vessel classification sensitivity confirmed that event timing affected interpretation. Restricting the 30-day endpoint to postprocedural loss of patency yielded a single-source estimate of 0/84 vessels. A broader analysis that also counted the index accessory renal incorporation failure in Csobay-Novák 2025 [22] yielded 0.8% (95% CI 0.1–5.4%; two populations, 127 vessels), whereas the distinct patient-level outcome during available follow-up was 2.4% (95% CI 0.6–9.1%; two populations, 83 patients). These results were not treated as interchangeable.

Leave-one-out estimates ranged from 96.6% to 98.2% for technical success, 1.1% to 2.7% for 30-day mortality, 3.2% to 5.0% for acute kidney injury, and 1.2% to 1.8% for stroke. In contrast, omission of either source changed the single-source remainder for any reintervention during available follow-up from 4.3% to 29.4%, confirming substantial source sensitivity. Any endoleak during available follow-up similarly ranged from 12.8% to 40.8% after source omission.

Acute kidney injury, excluding the inferred-zero row from Csobay-Novák 2025 [22], produced 4.7% (95% CI 2.7–8.1%; three populations, 254 patients), compared with 4.5% in the strict primary analysis. Including all reports without overlap adjustment produced broadly similar early estimates, but 30-day mortality increased from 2.3% to 3.9%; this analysis was secondary because overlapping UW IDE patients could contribute more than once. Device-related or aortic-related reintervention remained unsuitable for pooling because it was sparsely reported and inconsistently defined. Detailed sensitivity results are provided in Supplementary Table S4.

Prespecified subgroup analyses by IDE status, device platform, modification type, and number of target vessels were not retained in the main manuscript. The strict primary dataset yielded too few independent populations per subgroup, and the unrestricted dataset was dominated by overlapping single-institution reports (10 of 15 reports). The small number of independent populations also precluded formal small-study effect testing for primary endpoints.

## 4. Discussion

The present systematic review and single-arm meta-analysis identified a strong technical-feasibility signal for physician-modified endograft (PMEG) repair in selected patients with juxtarenal and pararenal abdominal aortic aneurysms. In the strict overlap- and pathology-adjusted analysis, technical success was 97.8%, 30-day mortality was 2.3%, acute kidney injury was 4.5%, stroke was 1.6%, and spinal cord ischemia was 1.7%. These estimates were informed by only two to four independent populations, and the certainty of evidence was very low. Follow-up report-period outcomes were even more sparse and heterogeneous. The results therefore support technical feasibility in experienced hands but do not establish clinical effectiveness, durability, equivalence, non-inferiority, or superiority relative to custom-manufactured fenestrated endovascular aneurysm repair (FEVAR).

Although endpoint-level overlap adjudication reduced duplicate patient counting, the evidence base remained dominated by overlapping reports from a single highly experienced PMEG program. The five independent non-UW reports were small, and the only five-patient report did not enter the strict primary pooling because its pathology was inseparable. Leave-one-out analyses did not materially change the main early estimates, but they cannot compensate for sparse external replication. The pooled estimates should therefore be interpreted as expert-program outcomes rather than average real-world performance.

The most clinically relevant benchmark for PMEG is custom-manufactured FEVAR. Both PMEG and custom-manufactured FEVAR aim to extend the proximal sealing zone while preserving renal and visceral perfusion in patients with inadequate infrarenal neck anatomy. Current guidelines and published FEVAR series support fenestrated repair as an established complex endovascular strategy for anatomically suitable juxtarenal and pararenal aneurysms, particularly in the elective setting when device planning and manufacturing time are clinically acceptable [2,25,26,27,28]. In the United States Zenith Fenestrated multicenter study, technical success was 100% and 30-day mortality was 1.5% [25]. A contemporary single-center FEVAR series reported technical success of 98%, 30-day mortality of 0.9%, 5-year target-vessel patency of 98.7%, and 5-year freedom from reintervention of 86.5% [27]. Long-term FEVAR data also show that reintervention and target-vessel surveillance remain important even after technically successful repair [28]. These data provide clinically relevant benchmarks for interpreting PMEG outcomes, but they should not be used as evidence of equivalence, non-inferiority, or superiority. The present review did not include head-to-head comparative studies between PMEG and custom-manufactured FEVAR, and the included PMEG cohorts differed from typical elective FEVAR cohorts in urgency, anatomy, device availability, and operator expertise.

The contrast with FEVAR should therefore be understood as a contrast between clinical roles rather than a direct comparison of treatment effects. In stable elective patients with suitable anatomy and sufficient time for device manufacture, custom-manufactured FEVAR remains the more standardized and evidence-supported strategy. PMEG becomes more relevant when this pathway is unsafe or impractical, such as in symptomatic aneurysms, contained or impending rupture, rapidly enlarging aneurysms, selected infected or mycotic aneurysms, type Ia endoleak with sac growth, proximal seal failure, or anatomically constrained patients in whom standard EVAR is unsuitable and open repair carries prohibitive risk. In these circumstances, the value of PMEG is not theoretical superiority over FEVAR, but immediate availability of an anatomically tailored complex endovascular option.

The rationale for PMEG is primarily related to availability, urgency, anatomy, and operative risk. Custom-manufactured fenestrated or branched devices require time for planning, production, delivery, and procedural scheduling. The earliest PMEG report noted that custom fenestrated grafts typically require 6 to 12 weeks to manufacture and that, even under favorable conditions, the interval was no shorter than 8 to 12 weeks [7]. Subsequent University of Washington reports described manufacturing delays of up to 12 weeks, whereas contemporary U.S. acquisition times of 6 to 8 weeks were noted in the long-term IDE report [13,14]. In a Swiss juxtarenal PMEG series, production times of up to 16 weeks were cited [20]. In a 906-patient planned F/BEVAR waiting-time series, the median interval from graft order to operation was 12 weeks, overall aneurysm rupture during the waiting period occurred in 18 patients (2.0%), and mortality during the waiting period was 4.1% [29]. Waiting may be acceptable for stable elective aneurysms but unsafe for symptomatic aneurysms, contained or impending rupture, rapidly expanding aneurysms, selected infected or mycotic aneurysms, type Ia endoleak with sac growth, proximal seal failure, or selected redo complex aortic pathology [29,30,31]. These indications were represented in several independent non-University of Washington reports, including symptomatic or impending/contained rupture and mycotic aneurysms in the Korean cohort, urgent rupture or symptomatic aneurysms and type Ia endoleak with sac growth in the German cohort, and non-deferrable complex abdominal aortic aneurysm repair in the Spanish multicenter experience [21,23,24]. In these settings, the value of PMEG is not theoretical superiority over FEVAR, but immediate availability of an anatomically tailored complex endovascular solution for patients in whom waiting for a custom-manufactured device is unsafe or impractical.

This indication should be defined narrowly. PMEG is best justified when repair is non-deferrable: contained or impending rupture, symptomatic aneurysm, rapidly enlarging aneurysm, selected infected or mycotic aneurysm, failed proximal seal after prior EVAR, type Ia endoleak with sac growth, or anatomically constrained patients in whom standard EVAR is unsuitable and open repair is judged prohibitively hazardous. The same logic may apply to selected redo or proximal anastomotic pseudoaneurysm cases when there is no suitable ready-made endovascular alternative. For stable elective patients, in contrast, the present data do not support PMEG as a routine substitute. PMEG should be considered a selective, time-sensitive strategy rather than a default elective pathway.

The favorable perioperative results observed in this review are technically plausible. PMEG outcomes depend on accurate centerline-based planning, reliable fenestration alignment, careful back-table modification, secure reinforcement, controlled resheathing, target-vessel catheterization, and high-quality intraoperative imaging. The maturation of these workflows is evident in reports describing automated planning, standardized templates, cautery or scalpel fabric openings, and several reinforcement methods [10,13,14,20,22,24]. The available literature does not, however, permit attribution of Type III endoleak to a specific fabric-opening tool. Type III events may arise from bridging-stent, component, alignment, or junctional failure, and method-stratified denominators were not reported consistently. The present data therefore support transparent technical reporting rather than a claim that one opening or reinforcement method is safer [32].

Postoperative endograft infection was similarly underreported. Open back-table exposure and manipulation of graft fabric may introduce a theoretical contamination risk, but the available reports did not permit comparison with unmodified or custom-manufactured devices. One presumed infection was described in the long-term UW IDE report and likely reappeared in an overlapping late subset; it should be considered one possible event rather than two independent infections [14,15]. Most reports did not define systematic infection surveillance, and shorter follow-up could miss delayed infection. The review therefore cannot estimate infection incidence or determine whether back-table modification changes infection risk. This uncertainty is distinct from the use of PMEG to treat pre-existing mycotic aneurysm or infectious penetrating aortic ulcer.

These technical requirements explain why generalizability is limited. PMEG is not a standardized commercial product but a complex operator-dependent strategy. Outcomes achieved by a mature program with extensive planning infrastructure, a dedicated team, and rigorous surveillance may not be reproduced during the learning curve or in lower-volume centers. High technical success in this review should thus be viewed as an expert-center benchmark rather than a universal expectation.

Aortic morphology also reflects an underlying biological substrate that is not captured by diameter and seal-zone measurements alone. Histopathological studies of aortic aneurysm and dissection describe medial, myxomatous, and subendothelial degenerative changes and emphasize heterogeneity in aortic-wall disease [33]. These data were derived mainly from surgically excised non-PMEG aortic specimens and do not provide direct evidence about PMEG durability. Progressive degeneration of the diseased sealing-zone aorta may nevertheless underlie late Type Ia endoleak, neck dilation, and migration after endovascular repair. These observations reinforce the need to interpret anatomy, remodeling, and surveillance longitudinally rather than assuming that initial technical sealing fully characterizes later risk.

The sensitivity analyses support this cautious interpretation. Restriction to explicit technical-success composites yielded 97.6%, and replacing Tachida 2023 with the percentage-derived Starnes 2024 estimate changed technical success from 97.8% to 94.3% while preserving a high feasibility signal [13,14]. Excluding the inferred-zero acute kidney injury row changed the estimate only minimally, from 4.5% to 4.7%. The broader mixed-pathology analysis also produced similar early estimates, whereas unrestricted inclusion of overlapping reports increased 30-day mortality to 3.9%. Distinguishing index target-vessel incorporation failure from subsequent occlusion changed the strict 30-day result from the submitted 0.8% pooled estimate to a single-source 0/84 estimate; patient-level occlusion during available follow-up was 2.4%. Any reintervention during available follow-up was highly source-sensitive: the strict pooled estimate was 13.2% with a 95% confidence interval of 2.9% to 43.7%, I^2^ of 89.0%, and single-source remainders of 4.3% and 29.4%. Feasibility appears more robust than the safety and follow-up signals.

Durability remains the major unresolved issue. A pooled 1-year analysis was not possible because the reports mixed Kaplan–Meier freedom-from-event estimates, incomplete risk sets, report-period event counts, and overlapping populations. Estimates during available follow-up therefore represent exploratory proportions over different observation periods, not cumulative-incidence estimates at a shared time point. Type III endoleak had only one strict source and was not pooled. Patient-level target-vessel occlusion could be reconstructed from two independent populations, but only 83 patients contributed and the 95% confidence interval ranged from 0.6% to 9.1%. Any endoleak and any reintervention were imprecise and heterogeneous, likely reflecting differences in surveillance intensity, follow-up duration, reintervention thresholds, definitions, and reporting practice.

Several additional sources of bias constrain interpretation. Denominator and definition heterogeneity are particularly relevant for renal and target-vessel outcomes because some reports separated main from accessory renal incorporation and used vessel-level technical or patency outcomes, whereas others emphasized patient-level morbidity [20,24,32]. No report provided complete outcomes for all urgent patients assessed but deemed unsuitable for PMEG, so the treated cohorts cannot define the risk among all urgent candidates. Publication and reporting bias could not be excluded because unsuccessful modifications, aborted procedures, screened-but-untreated patients, delayed infections, and early learning-curve outcomes may be less likely to appear in small expert-center reports.

The distinction between methodological reporting quality and certainty of evidence is important. The JBI appraisal described report-level methodological quality, including inclusion criteria, patient characterization, consecutive inclusion, outcome measurement, and follow-up completeness [17]. It did not convert 15 reports into 15 independent cohorts and should not be interpreted as evidence certainty. GRADE was assessed separately at the outcome level and remained very low for every key outcome [18]. This rating reflects noncomparative design, imprecision, expert-center indirectness, sparse independent populations, possible publication bias, and, for reintervention, substantial inconsistency. Methodologically well-described case series can still provide very uncertain comparative or generalizable evidence.

Several limitations should be considered. The evidence consisted entirely of nonrandomized single-arm observational case series, precluding direct comparative inference against custom-manufactured FEVAR, off-the-shelf branched devices, chimney techniques, or open repair. Endpoint-level overlap adjudication reduced duplicate counting but could not remove expert-program dominance. Strict pathology adjudication reduced the external evidence further, and the minimum report size of five remained vulnerable to unstable estimates. Definitions and follow-up were inconsistent, infection and screened-but-untreated urgent candidates were incompletely reported, and vessel-level perioperative outcomes were not interchangeable with patient-level follow-up risk. Some zero-event data required cautious inference from narrative reporting. These constraints limit conclusions regarding effectiveness, durability, and broader generalizability.

The clinical implication is not that PMEG should replace custom-manufactured FEVAR, but that it should be concentrated in audited high-volume aortic programs as a selective option for non-deferrable, anatomy-driven repair. PMEG use requires established complex aortic expertise, standardized planning and modification workflows, multidisciplinary selection, rescue capability, and lifelong imaging surveillance. A PMEG-specific extension of existing Society for Vascular Surgery reporting standards is needed [32]. At minimum, future reports should state all screened, excluded, initiated, completed, and converted cases; urgency and pathology by patient; explicit procedural and 30-day technical-success components; device platform, fabric-opening, reinforcement, bridging-stent, and resheathing methods; patient- and vessel-level denominators; postoperative infection surveillance; surveillance completeness; fixed-time and time-to-event endoleak, target-vessel, and reintervention outcomes; and patient-level identifiers or registry linkage sufficient for de-duplication. Prospective multicenter registries and comparative studies will be necessary to distinguish a reproducible treatment effect from a high-performance expert-center effect.

## 5. Conclusions

PMEG repair for patients with juxtarenal and pararenal abdominal aortic aneurysms demonstrated high technical feasibility and low observed early event rates in selected patients treated at experienced centers. Clinical effectiveness and durability remain uncertain because the certainty of evidence is very low, independent populations are sparse, and the evidence base is strongly influenced by one expert program. PMEG should be considered a specialized option for non-deferrable or anatomically constrained cases rather than a routine replacement for custom-manufactured FEVAR.

## Figures and Tables

**Table 1:** Characteristics of eligible reports.

First Author, Year	Country/ Center	Study Design	Evidence Source/ Overlap Category	N	Aneurysm/ Pathology Type	Urgency/ Indication	Age, y	Male, %	Modified Endograft Platform	Target Vessels/ Fenestrations	Follow- Up, Months
Starnes 2012 [7]	USA/University of Washington	Retrospective	UW pre-IDE independent cohort	47	JRAAA	Elective Symptomatic or Ruptured	75.0 (mean)	57.4%	Cook Zenith Flex	RRA/LRA/SMA/CA/accessory RA mean 1.7 per patient	19.9 (mean)
Starnes 2013 [8]	USA/University of Washington	Prospective	UW IDE early serial report	26	JRAAA	Elective Symptomatic or Ruptured	76.0 (mean)	73.1%	Cook Zenith Flex	RRA/LRA/SMA mean 2.5 per patient	11.9 (mean)
Starnes 2017 [9]	USA/University of Washington	Prospective	UW IDE midterm serial report	59	JRAAA	Elective Symptomatic or Ruptured	74.8 (mean)	76.0%	Cook Zenith Flex	RRA/LRA/SMA mean 2.5 per patient	27.6 (mean)
Starnes 2018 [10]	USA/University of Washington	Retrospective	UW IDE automated planning subset	30	JRAAA	Symptomatic Asymptomatic JRAAA	74.0 (mean)	70.0%	Mixed/other	RRA/LRA/SMA mean 2.9 per patient	NR
Qaderi 2019 [11]	USA/University of Washington	Retrospective	UW IDE imaging subset analysis	77	JRAAA	Asymptomatic Ruptured JRAAA	74.0 (mean)	75.0%	Mixed (Cook/Medtronic/Bolton)	RRA/LRA/SMA mean 2.5 per patient	12.0 (median)
Hemingway 2021 [12]	USA/University of Washington	Retrospective	UW IDE device-specific subset	46	JRAAA	Elective 100.0%	75.0 (mean)	70.0%	Terumo Treo	RRA/LRA/SMA mean 2.8 per patient	19.6 (mean)
Chan 2023 [20]	Switzerland/single center	Retrospective	Non-UW independent report	37	JRAAA	Elective 91.9% Urgent or emergent 8.1%	75.0 (median)	89.0%	Medtronic	Main/accessory RAs ± SMA mean 1.3 per patient	17.7 (median)
Tachida 2023 [13]	USA/University of Washington	Prospective	UW IDE reintervention focused analysis	170	JRAAA	Elective 94% Urgent 5% Emergent 1%	76.0 (mean)	78.0%	Mixed (Cook/Medtronic/Terumo)	RRA/LRA/SMA ± CA mean 2.9 per patient	33.0 (mean)
Kim 2024 [21]	Korea/single center	Retrospective	Non-UW independent report	12	10 JRAAA; 1 infrarenal AAA; 1 TAAA	Symptomatic 50.0% Impending or ruptured 25.0% Mycotic 16.7% Other 8.3%	74.0 (mean)	91.7%	Cook Zenith Flex	CA/SMA/RRA/LRA mean 2.1 per patient	32.3 (mean)
Starnes 2024 [14]	USA/University of Washington	Prospective	UW IDE comprehensive long-term report	203	JRAAA	Elective Symptomatic ruptured	75.6 (mean) *	77.0% *	Mixed (Terumo/Cook/Medtronic/Bolton)	RRA/LRA/SMA/CA mean 2.8 per patient	NR
Csobay-Novák 2025 [22]	Hungary/single center	Prospective	Non-UW independent report	11	JRAAA/ PRAAA	Symptomatic 18.2%	76.0 (mean)	72.7%	Terumo Treo	RRA/LRA/SMA/CA mean 3.9 per patient	NR
Lescan 2025 [23]	Germany/single center	Single-center observational study	Non-UW independent report	5	1 PRAAA; 2 post-EVAR Type Ia failures; 2 PAUs	Ruptured symptomatic type Ia endoleak with growth	82.0 (median)	60.0%	Terumo Treo	RRA/LRA/SMA/CA mean 2.6 per patient	NR
Murphy 2025 [15]	USA/University of Washington	Retrospective analysis	UW IDE >5-year cause of death subset	98	JRAAA	Elective 90.9% Symptomatic 7.1% Ruptured 1.0% Other 1.0%	74.0 (mean)	76.5%	Mixed/other	RRA/LRA/SMA mean 3.0 per patient	59.4 (median)
Nelson 2025 [16]	USA/University of Washington	Retrospective analysis	UW IDE anatomical predictor subset	142	JRAAA	NR	NR	NR	Mixed (Cook/Medtronic/Terumo/Bolton)	Predominantly three-vessel fenestration median 3 per graft	64.4 (mean)
Álvarez Marcos 2025 [24]	Spain/multicenter	Retrospective	Non-UW independent report	18	6 JRAAA/PRAAA; 6 no-neck AAA; 6 post-EVAR Type Ia failures	Symptomatic 33.3%	79.4 (mean)	94.0%	Medtronic Endurant	RRA/LRA/SMA/CA mean 2.1 per patient	10.0 (median)

Note: This table summarizes eligible reports rather than independent patient populations. Reported N refers to the sample size described in each publication before overlap adjudication. Starnes 2012 [7] was treated as an independent pre-IDE University of Washington cohort. Reports published from 2013 onwards from the University of Washington/Harborview PMEG program were considered serial, subset, or endpoint-specific analyses of the UW IDE/NCT01538056 cohort. Kim 2024, Lescan 2025, and Álvarez Marcos 2025 were retained as eligible mixed-pathology reports but excluded from strict primary pooling because target-population endpoint denominators could not be separated [21,23,24]. Endpoint-level data use and overlap management are detailed in Supplementary Table S2. NR indicates not reported in the original article; missing report-level characteristics were not imputed. Modified endograft platform refers to the main aortic endograft used for physician modification. Bridging stents, reinforcement materials, and resheathing techniques are provided in Supplementary Table S6 and the complete extracted dataset. * Baseline demographic values in Starnes 2024 [14] were reported for screened subjects who completed the screening process, whereas the reported N refers to 203 patients who received PMEG. The source article reported 30-day all-cause mortality as 6/204; this endpoint-specific denominator was retained in the mortality meta-analysis. Abbreviations: AAA, abdominal aortic aneurysm; CA, celiac artery; EVAR, endovascular aneurysm repair; IDE, investigational device exemption; JRAAA, juxtarenal abdominal aortic aneurysm; LRA, left renal artery; NR, not reported; PAU, penetrating aortic ulcer; PRAAA, pararenal abdominal aortic aneurysm; RA, renal artery; RRA, right renal artery; SMA, superior mesenteric artery; TAAA, thoracoabdominal aortic aneurysm; UW, University of Washington.

**Table 2:** Primary overlap- and pathology-adjusted results.

Outcome	Time Point	Independent Populations/Denominator	Estimate, % (95% CI)	I^2^ (%)
Technical success	Perioperative/30 days	3/228	97.8% (94.8–99.1%)	4.0%
30-day mortality	30 days	4/299	2.3% (1.1–4.8%)	0.0%
Acute kidney injury	30 days	4/265	4.5% (2.6–7.8%)	0.0%
Stroke	30 days	3/254	1.6% (0.6–4.1%)	0.0%
Spinal cord ischemia	30 days	2/181	1.7% (0.5–5.0%)	0.0%
Type Ia endoleak	30 days	2/41	0.0% (0.0–8.6%)	Not estimable
Type III endoleak	30 days	2/41	0.0% (0.0–8.6%)	Not estimable
Any endoleak	30 days	2/70	17.1% (10.0–27.8%)	0.0%
Target-vessel occlusion, vessel-level	30 days	1/84 vessels	Not pooled; 0.0% (0.0–4.3%)	Not estimable
Type Ia endoleak	During available follow-up (exploratory)	2/250	0.8% (0.2–3.1%)	7.4%
Type III endoleak	During available follow-up (exploratory)	1/203	Not pooled; 3.4% (1.4–7.0%)	Not estimable
Any endoleak	During available follow-up (exploratory)	2/145	25.3% (10.1–50.3%)	90.3%
Any reintervention	During available follow-up (exploratory)	2/217	13.2% (2.9–43.7%)	89.0%
Target-vessel occlusion, patient-level	During available follow-up (exploratory)	2/83	2.4% (0.6–9.1%)	0.0%

Note: The strict primary analysis was performed at the level of independent target-population cohorts rather than individual publications. Starnes 2012 [7] was treated as an independent pre-IDE University of Washington cohort. For the subsequent UW IDE/NCT01538056 cluster, no more than one report was allowed to contribute data to any endpoint. Kim 2024, Lescan 2025, and Álvarez Marcos 2025 [21,23,24] were excluded from strict pooling because target JRAAA/PRAAA endpoint denominators were not separable. Thirty-day target-vessel occlusion used a vessel-level denominator; target-vessel occlusion during available follow-up used patient-level denominators. During-follow-up, estimates are exploratory report-period proportions and not common fixed-time cumulative incidences. Single-source endpoints were not pooled. Endpoint-level source selection, events, denominators, and exclusions are detailed in Supplementary Table S2; GRADE certainty is presented in Supplementary Table S3. Abbreviations: CI, confidence interval; GRADE, Grading of Recommendations Assessment, Development and Evaluation; IDE, investigational device exemption; I^2^, Higgins–Thompson heterogeneity statistic; JRAAA, juxtarenal abdominal aortic aneurysm; PMEG, physician-modified endograft; PRAAA, pararenal abdominal aortic aneurysm.

## Data Availability

No new individual participant data were generated in this study. All data supporting the findings were extracted from published articles and are provided in the Supplementary Materials, including Supplementary Data File S1 and Supplementary Tables S1–S7. Derived analysis manifests and sensitivity results are included in these files; additional analysis code is available from the corresponding author upon reasonable request.

## Supplementary Materials

The following supporting information can be downloaded at https://www.mdpi.com/article/10.3390/jcm15176578/s1: Supplementary File S1: PRISMA 2020 checklist; Supplementary File S2: search strategy; Supplementary Table S1: full-text exclusions; Supplementary Table S2: overlap adjudication and endpoint-source manifest; Supplementary Table S3: GRADE evidence profile; Supplementary Table S4: sensitivity analyses; Supplementary Table S5: JBI checklist; Supplementary Table S6: technical definitions and device modification; Supplementary Table S7: pathology composition and quantitative eligibility; Supplementary Data File S1: complete extraction dataset.
