# Supplementary material for: Outcomes of Physician-Modified Endografts for Juxtarenal and Pararenal Abdominal Aortic Aneurysms: A Systematic Review and Single-Arm Meta-Analysis
Source: J Clin Med. 2026 Aug 26;15(17):6578. doi: 10.3390/jcm15176578 (PMC13566218; doi:10.3390/jcm15176578)
Supplement: Supplementary file 1 [file jcm-15-06578-s001.zip › Supplementary File S2 Search_strategy.pdf]

Supplementary: Search Strategy

Search date: December 26, 2025. No language or date restrictions were applied.

1. PubMed (n = 770)

```
(
  ("physician-modified"[tiab] OR "physician modified"[tiab]
   OR "surgeon-modified"[tiab] OR "surgeon modified"[tiab]
   OR "operator-modified"[tiab] OR "operator modified"[tiab]
   OR PMEG[tiab] OR PMSG[tiab]
   OR ("modified"[tiab] AND (endograft*[tiab] OR "stent graft*"[tiab]
       OR stentgraft*[tiab]))
  )
  AND
  (fenestrat*[tiab] OR "fenestrated"[tiab] OR "fenestration"[tiab]
   OR branch*[tiab] OR "branched"[tiab]
   OR endograft*[tiab] OR "stent graft*"[tiab] OR stentgraft*[tiab]
   OR endovascular[tiab]
  )
  AND
  (aort*[tiab] OR aneurysm*[tiab] OR "abdominal aortic aneurysm"[tiab]
   OR "aorta"[MeSH] OR "aortic aneurysm, abdominal"[MeSH]
  )
)
NOT (animals[MeSH] NOT humans[MeSH])
```

2. Embase via Ovid (n = 959)

```
1. (physician-modified OR "physician modified" OR surgeon-modified
   OR "surgeon modified" OR operator-modified OR "operator modified"
   OR PMEG OR PMSG
   OR (modified NEAR/3 (endograft* OR "stent graft*"
       OR stentgraft*))):ti,ab,kw

2. (fenestrat* OR fenestrated OR fenestration OR branch* OR branched
   OR endograft* OR "stent graft*" OR stentgraft*
   OR endovascular):ti,ab,kw

3. (juxtarenal OR pararenal OR para-renal OR "short neck"
   OR "complex abdominal" OR "complex aaa"):ti,ab,kw

4. ((aort* OR "abdominal aortic aneurysm" OR AAA OR aneurysm*
   OR "aorta") OR (abdominal AND aort*)):ti,ab,kw

5. #1 AND #2 AND (#3 OR #4)
```

3. Web of Science Core Collection (n = 573)

```
("physician modified" OR "physician-modified"
 OR "surgeon modified" OR "surgeon-modified"
 OR "operator modified" OR "operator-modified"
 OR PMEG OR PMSG
 OR (modified NEAR/3 (endograft* OR "stent graft*" OR stentgraft*)))
AND
(fenestrat* OR fenestrated OR fenestration OR branch* OR branched
 OR endograft* OR "stent graft*" OR stentgraft* OR endovascular)
AND
```

```
(juxtarenal OR pararenal OR "para renal" OR "para-renal"
 OR "short neck" OR "complex abdominal" OR "complex AAA"
 OR (abdominal NEAR/3 aort*) OR aort*)
```

4. Cochrane Library (n = 1)

```
("physician-modified" OR "surgeon-modified"
 OR PMEG OR PMSG
 OR (modified NEXT endograft)
 OR (modified NEXT "stent graft"))
AND
(fenestrat* OR fenestrated OR fenestration OR endograft*
 OR "stent graft*" OR endovascular)
AND
(juxtarenal OR pararenal OR "short neck" OR abdominal OR aort*)
```

5. ClinicalTrials.gov (n = 25)

A supplementary search of ClinicalTrials.gov was performed using the terms "physician-modified endograft," "PMEG," "surgeon-modified endograft," and "fenestrated endovascular" to identify registered clinical trials with potentially extractable outcome data.

Summary of database yields

Database	Records identified
PubMed	770
Embase via Ovid	959
Web of Science Core Collection	573
Cochrane Library	1
Subtotal (databases)	2,303
ClinicalTrials.gov	25
Total all sources	2,328
Duplicates removed (databases)	1,033
Unique records screened	1,270
