# Supplementary material for: Outcomes of Physician-Modified Endografts for Juxtarenal and Pararenal Abdominal Aortic Aneurysms: A Systematic Review and Single-Arm Meta-Analysis
Source: J Clin Med. 2026 Aug 26;15(17):6578. doi: 10.3390/jcm15176578 (PMC13566218; doi:10.3390/jcm15176578)
Supplement: Supplementary file 1 [file jcm-15-06578-s001.zip › Supplementary_Table_S3_GRADE_evidence.pdf]

Supplementary Table S3. GRADE certainty assessment for key single-arm outcomes
Author(s): Review authors
Question: Physician-modified endograft repair for patients with juxtarenal or pararenal abdominal aortic aneurysms.
Setting: Single-arm observational evidence from specialist vascular centres performing complex endovascular aortic repair.
Bibliography: Included reports are listed in the main reference list and Supplementary Table S2.

Certainty assessment							No. of patients		Effect		Certainty	Importance
N	Study design	Risk of bias	Inconsistency	Indirectness	Imprecision	Other considerations	Physician-modified endograft repair	Not applicable[1]	Relative (95% CI)[1]	Absolute (95% CI)		
**Technical success[7]**												
3	non-randomised studies	Serious[2]	not serious	Serious[3]	Serious[4]	publication bias could not be excluded[5]	223/228 (97.8%)	—	not estimable	97.8% (95% CI, 94.8–99.1%)	⊕◯◯◯ Very low	Critical
**30-day mortality**												
4	non-randomised studies	serious[2]	not serious	serious[3]	serious[4]	publication bias could not be excluded[5]	7/299 (2.3%)	—	not estimable	2.3% (95% CI, 1.1–4.8%)	⊕◯◯◯ Very low	Critical
**Acute kidney injury**												
4	non-randomised studies	serious[2]	not serious	serious[3]	serious[4]	publication bias could not be excluded[5]	12/265 (4.5%)	—	not estimable	4.5% (95% CI, 2.6–7.8%)	⊕◯◯◯ Very low	Critical
**Spinal cord ischemia**												
2	non-randomised studies	serious[2]	not serious	serious[3]	serious[4]	publication bias could not be excluded[5]	3/181 (1.7%)	—	not estimable	1.7% (95% CI, 0.5–5.0%)	⊕◯◯◯ Very low	Critical
**Type III endoleak during available follow-up**												
1	non-randomised studies	serious[2]	not serious	serious[3]	very serious[4]	publication bias could not be excluded[5]	7/203 (3.4%)	—	not estimable	3.4% (95% CI, 1.4–7.0%); not pooled	⊕◯◯◯ Very low	Critical
**Target-vessel occlusion during available follow-up, patient-level**												
2	non-randomised studies	serious[2]	not serious	serious[3]	very serious[4]	publication bias could not be excluded[5]	2/83 patients (2.4%)	—	not estimable	2.4% (95% CI, 0.6–9.1%)	⊕◯◯◯ Very low	Critical
**Any reintervention[8]**												
2	non-randomised studies	serious[2]	serious	serious[3]	very serious[4]	publication bias could not be excluded[5]	52/217 (24.0%)	—	not estimable	13.2% (95% CI, 2.9–43.7%)	⊕◯◯◯ Very low	Critical

Certainty assessment							No. of patients		Effect		Certainty	Importance
N	Study design	Risk of bias	Inconsistency	Indirectness	Imprecision	Other considerations	Physician-modified endograft repair	Not applicable[1]	Relative (95% CI)[1]	Absolute (95% CI)		
**Stroke**												
3	non-randomised studies	serious[2]	not serious	serious[3]	serious[4]	publication bias could not be excluded[5]	4/254 (1.6%)	—	not estimable	1.6% (95% CI, 0.6–4.1%)	⊕◯◯◯ Very low	Critical

CI: confidence interval; PMEG: physician-modified endograft

Explanations

1. No direct comparator was available. Certainty was assessed for pooled single-arm proportions rather than comparative effect estimates.

2. Downgraded for serious risk of bias because the evidence consisted of non-randomised single-arm series; patient selection was determined by operator and institutional preference, and no adjustment for confounding by indication was possible.

3. Downgraded for indirectness because the evidence was dominated by highly selected expert-centre PMEG cohorts, limiting generalisability to routine practice and lower-volume centres.

4. Downgraded for imprecision because only one to four independent patient populations informed each strict target-population estimate and confidence intervals were wide for several outcomes.

5. Publication bias could not be excluded because the evidence base consisted mainly of small single-centre and expert-centre reports; formal small-study effect testing was not performed because no outcome included at least 10 independent populations.

6. Thirty-day target-vessel occlusion was available only at the vessel level. During available follow-up, the endpoint was analysed at the patient level using reports that stated the number of patients with at least one occluded target vessel.

7. The strict target-population technical-success analysis retained Starnes 2012, Tachida 2023, and Csobay-Novak 2025. Definitions shared core deployment and target-vessel components but were not identical. A sensitivity analysis restricted to explicit composite definitions yielded 97.6% (95% CI, 94.3–99.0%); replacement with percentage-derived Starnes 2024 data yielded 94.3% (95% CI, 90.7–96.5%).

8. Any reintervention during available follow-up was an exploratory report-period proportion and was downgraded for inconsistency because substantial heterogeneity was observed ($I^2 = 89.0\%$), likely reflecting differences in follow-up duration, endpoint definitions, and reintervention thresholds.
